# A possible candidate for triply degenerate point fermions in trigonal layered PtBi_2_

**DOI:** 10.1038/s41467-018-05730-3

**Published:** 2018-08-14

**Authors:** Wenshuai Gao, Xiangde Zhu, Fawei Zheng, Min Wu, Jinglei Zhang, Chuanying Xi, Ping Zhang, Yuheng Zhang, Ning Hao, Wei Ning, Mingliang Tian

**Affiliations:** 1 Anhui Province Key Laboratory of Condensed Matter Physics at Extreme Conditions, High Magnetic Field Laboratory, Chinese Academy of Sciences, Hefei, 230031 China; 2 Department of physics, University of Science and Technology of China, Hefei, 230026 China; 3 Institute of Physical Science and Information Technology, School of Physics and Materials Science, Anhui University, Hefei, 230601 China; 4 Institute of Applied Physics and Computational Mathematics, Beijing, 100088 China; 5 Beijing Computational Science Research Center, Beijing, 100193 China; 6 Collaborative Innovation Center of Advanced Microstructures, Nanjing University, Nanjing, 210093 China

## Abstract

Triply degenerate point (TP) fermions in tungsten–carbide-type materials (e.g., MoP), which represent new topological states of quantum matter, have generated immense interest recently. However, the TPs in these materials are found to be far below the Fermi level, leading to the TP fermions having less contribution to low-energy quasiparticle excitations. Here, we theoretically predict the existence of TP fermions with TP points close to the Fermi level in trigonal layered PtBi_2_ by ab initio calculations, and experimentally verify the predicted band topology by magnetotransport measurements under high magnetic fields up to 40 T. Analyses of both the pronounced Shubnikov–de Haas and de Haas–van Alphen oscillations reveal the existence of six principal Fermi pockets. Our experimental results, together with those from ab initio calculations, reveal the interplay between transport behaviors and unique electronic structures, and support the existence of TP fermions in trigonal layered PtBi_2_.

## Abstract (web summary)

Triply degenerate point (TP) fermions have been reported in MoP but the TPs are far below the Fermi level. Here, Guo et al. predict and verify the possible existence of TP fermions in trigonal layered PtBi_2_, where the TP points are close to the Fermi level.

## Introduction

As the counterparts of elementary particles of the standard model, the Dirac^1–4^ and Weyl^5–10^ fermions are found to exist in the form of quasiparticle excitations near Dirac and Weyl points, respectively. In Dirac semimetals, the Dirac points are fourfold degenerate with linear energy dispersion near the points^11–13^. By breaking either time-reversal or spatial inversion symmetry, one Dirac point splits into two Weyl points with twofold degeneracy^14–17^. Recently, several new fermions, which have no elementary particle counterparts, have been theoretically predicted as quasiparticle excitations near certain band-crossing points that are protected by several space-group symmetries^18,19^. In particular, triply degenerate point (TP) fermions have been predicted to exist in the electronic structure of a series of materials with tungsten–carbide (WC-) type crystal structure^20–23^. TPs can be viewed as an intermediate state between fourfold degenerate Dirac points and twofold degenerate Weyl points. Recent angle-resolved photoemission spectroscopy (ARPES) measurements have demonstrated the presence of TPs in MoP^24^ and WC^25^. Several magnetotransport studies on the WC-type materials, i.e., MoP^26^, WC^27^, and ZrTe^28^ have also been reported. However, the TP fermions are thought to make little contribution to the low-energy quasiparticle excitations because the TPs in MoP are far below the Fermi level (~1.5 eV). As a consequence, the exotic transport phenomena ascribed to TP fermions are difficult to test. Therefore, searching for new materials with TPs close to the Fermi level is essential to explore the exotic physics.

In this work, we theoretically predict that the trigonal layered PtBi_2_ hosts TP fermions that are close to the Fermi level. We performed angle-dependent magnetoresistance (MR) measurements under magnetic fields up to 40 T, and observed pronounced the Shubnikov–de Haas (SdH) quantum oscillations with a gigantic MR greater than 1.3 × 10^5^% without any sign of saturation at 32 T and 1.8 K when the magnetic field is tilted approximately 30° from the *c*-axis to the *a*-axis in the *ac* plane. Similar oscillations in magnetization are also observed clearly due to the de Haas–van Alphen (dHvA) effect. Analyses of both SdH and dHvA oscillations reveal the existence of six principal Fermi pockets, in which three present signatures of relativistic fermion behavior with light effective masses. Combining the results of ab initio calculations and theoretical analyses, we demonstrate that the exotic experimental results can be reasonably understood within the framework of unique electronic structures around the TPs.

## Results

### Crystal structure and schematic electronic structures

**Fig. 1:**
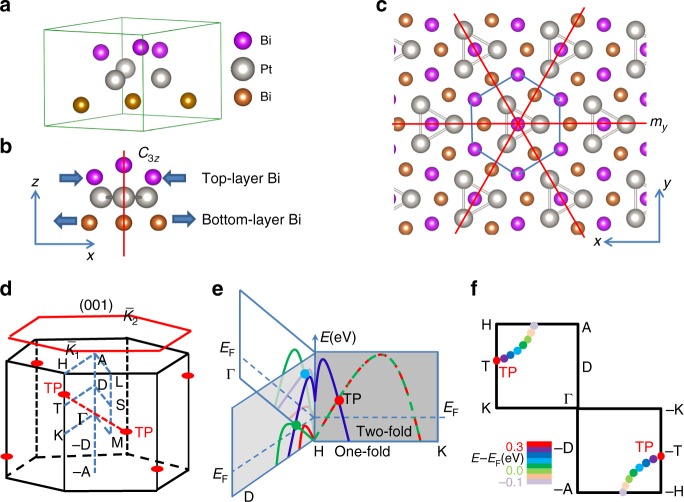
Crystal structure and schematic electronic structure. **a** Three-dimensional crystal structure of trigonal layered PtBi_2_. Top and bottom layers of Bi are labeled with different colors. **b** Side view of the lattice showing *C*_3*z*_ rotation symmetry. Shrinking of top Bi layer and extension of bottom Bi layer result in a lack of an inverse center of trigonal layered PtBi_2_. **c** Top view of lattice showing *m*_*y*_ mirror symmetry; other two mirror reflection symmetries are obtained by rotating the top Bi atom. **d** Three-dimensional bulk Brillouin zone (BZ) and (001) surface BZ with high-symmetry points are indicated. Red spots indicate TPs. Red-dashed line connects a pair of TPs. **e** Schematic band structures along three different high-symmetry lines. Red spots at the crossing points along line H–K indicate the TPs. The light blue and green spots along H–Г and H–D are doubly degenerate points. Curves of mixed red and green color represent doubly degenerate bands (twofold) and those of single color represent nondegenerate bands (onefold). **f** Tracks of degenerate point around the Fermi level in H–K–Γ–A plane. Colors of the spots represent the energy relative to the Fermi energy *E*_F_. The point at line H–K is a TP, and the points off the H–K line are doubly degenerate

Trigonal layered PtBi_2_ can be also denoted Bi_2_Pt (*hP*9), which was experimentally reported in ref. ^29^. Our ab initio calculations using the crystal structure analysis by particle swarm optimization algorithm have identified that trigonal layered PtBi_2_ with space-group *P*31*m* (No. 157) has the lowest-energy structure among a series of PtBi_2_ compounds (See Supplementary Figures 1 and 12 and Supplementary Table 1). The crystal structure lacks mirror symmetry *m*_*z*_ and inversion symmetry *i* due to the shrinking distortion of the top Bi layer and extension distortion of the bottom Bi layer, as shown in Fig. 1a. It also possesses rotational symmetry *C*_3*z*_ and mirror symmetry *m*_*y*_ [see Fig. 1b, c]. The main features of the electronic structures from the ab initio calculations are schematically illustrated in Fig. 1d–f. There exists a pair of TPs along the *C*_3*z*_ rotational axis H–K and (−H)–(−K) as shown in Fig. 1f, and they connect with each other by time-reversal symmetry, i.e., the two red dots connected by the red-dashed line in Fig. 1d. The band structures near one TP are shown in Fig. 1e. Apart from the H–K line in the *m*_*y*_ mirror plane, i.e., the H–K–Γ–A plane, the twofold degenerate bands split into two separate bands, which cross with the onefold band to form the linearly dispersive cross-points indicated by the colored spots in Fig. 1e, f. The cross-points form a track with different energy in the mirror plane in Fig. 1f. The features of the band structures can be roughly understood from symmetry arguments. For any point along the H–K line, the point group is *C*_3*v*_, which includes one unit operation, two threefold rotations, and three mirrors, i.e., {*E*, 2C_2*z*_, 3*m*}. The irreducible representations include one twofold *E* band and two onefold *A*_1_ and *A*_2_ bands. The ab initio calculations show that the twofold and onefold bands along the H–K line in Fig. 1e belong to the *E* and *A*_1_ irreducible representations, respectively. This is the reason they cross without opening a band gap, and the TPs are robust only if the symmetries in the *C*_3*v*_ point group are not broken. Apart from the H–K line, for example, along the H–D line as shown in Fig. 1e, the C_3*z*_ symmetry is broken, and the twofold bands have split into two bands with each band crossing with the onefold band to form doubly degenerate points. The doubly degenerate point is also robust and protected by mirror symmetry. More detailed analyses are shown below. The most remarkable feature of the electronic structure is that the energy window of the all cross-points including doubly and triply degenerate points lies in the range 0–0.3 eV when the Fermi level is set to 0 eV.

### Effect of magnetic field on resistivity

**Fig. 2:**
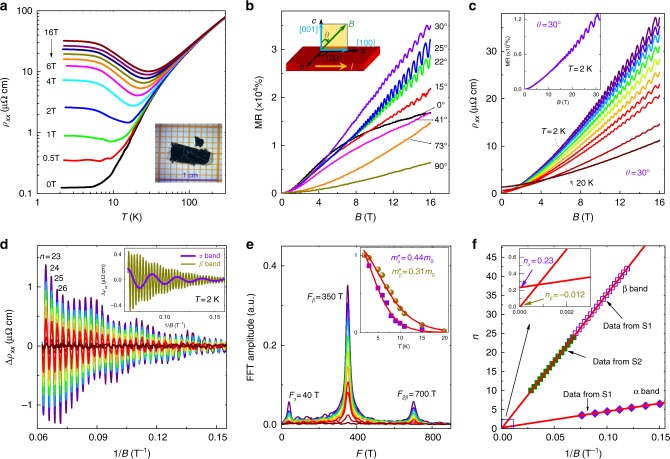
Temperature-dependent resistivity and SdH oscillations in magnetic fields up to 16 T. **a** Temperature dependence of the resistivity *ρ*_*xx*_ measured under different magnetic fields. Inset: Optical image of a typical single crystal. **b** Magnetic field dependence of MR under different angles *θ* at 2 K. *θ* changes from 0° to 90°, which corresponds to the magnetic field tilting from the [001] to [100] direction. **c** Magnetic field dependence of resistivity at different temperatures when the field is applied along *θ* = 30°. Inset: Magnetic field dependence of MR measured at 32 T and 2 K. **d** SdH oscillatory component as a function of 1/*B* after subtracting a smooth background. Inset: Two frequency components *F*_*α*_ = 40 T and *F*_*β*_ = 350 T extracted from oscillation patterns in the main panel. **e** Corresponding FFT spectra of the oscillations in the magnetic field range 8–16 T. Inset: Temperature dependence of the FFT amplitude. The solid curves are the fits to the LK formula. **f** Landau-level (LL) indices extracted from SdH oscillation plotted as a function of 1/*B*

To experimentally characterize the topological properties related with the TP fermions in this compound, we synthesized high-quality single crystals of trigonal layered PtBi_2_ [see inset of Fig. 2a] by the metallic flux method. The lattice parameters are experimentally determined as *a* = *b* = 6.571 Å, and *c* = 6.159 Å. Figure 2a shows the temperature dependence of resistivity of PtBi_2_ at various magnetic fields applied along the *c*-axis from 2 to 300 K. The zero-field resistivity exhibits highly metallic behavior with *ρ*_*xx*_(300 K) = 76.8 μΩ cm and *ρ*_*xx*_(2 K) = 0.12 μΩ cm; thus, the residual resistance ratio (*RRR*) reaches *R*(300 K)/*R*(2 K) = 640, which is significantly higher than that previously reported in the literature^30,31^, revealing the high quality of our samples. When a magnetic field is applied along the *c*-axis, the resistivity increases and shows a plateau at low temperature. Similar behaviors have been observed in other semimetals, such as Bi^32^, WTe_2_^33^, NbP^34^, and LaSb^35^.

Figure 2(b) shows the MR properties measured at 2 K with magnetic fields up to 16 T using a physical property measurement system (PPMS, Quantum Design, Inc.) oriented at different angles, *θ*, tilted from the *c*-axis ([001]) to the [100] direction. It was seen that the MR presents strong angle-dependent behavior. When the applied magnetic field is oriented along the direction at approximately *θ* = 30°, the unsaturated MR, [(*ρ*(*H*)−*ρ*(0))/*ρ*(0)] × 100% is as large as 4.0 × 10^4^ % at 16 T and ~1.3 × 10^5^ % at 32 T [see the inset of Fig. 2c], and exhibits quasilinear field dependence, accompanied with significant the SdH quantum oscillations. These properties have recently been observed in topological semimetals with linear Dirac dispersion^36,37^. When the field orientation is away from *θ* = 30°, the sample shows a smaller MR with a quadratic behavior and weak quantum oscillations. The angle-dependent MR, as shown in Supplementary Fig. 2b, exhibits an unusual butterfly-shaped pattern, indicating the strong anisotropy of the electronic band structures. Detailed discussions about such a unique angle dependence of MR and SdH oscillations are presented in the Supplementary information.

### Magnetoresistance oscillations

The strong SdH oscillations and giant linear MR around *θ* = 30° allow us to determine the nature of the band structures. Figure 2c shows the field dependence of resistivity measured at different temperatures with magnetic field *B* fixed along the direction *θ* = 30°. After subtracting a three-order polynomial background, the relative oscillatory component Δ*ρ*_*xx*_ versus 1/*B* is displayed in Fig. 2d. The Fast Fourier Transform (FFT) spectra of the oscillations in the magnetic field range 8–16 T are shown in Fig. 2e. The spectra reveal two principal frequencies, *F*_*α*_ = 40 T and *F*_*β*_ = 350 T,with a harmonic frequency *F*_2*β*_ = 700 T. According to the Onsager relation *F* = (ℏ/2*πe*)*A*_*F*_, where *F* is the frequency of oscillations, the cross-section of the Fermi surface, *A*_*F*_, is determined to be ~0.0038 and ~0.0331 Å^−2^ for *α* and *β* bands, respectively. The corresponding Fermi wave vector values *k*_*F*_ are ~0.035 and ~0.103 Å^−1^, respectively.

Generally, the effective mass m* can be obtained from the fit of the temperature dependence of the FFT amplitude of the SdH oscillations. For a system with arbitrary band dispersions, the amplitude of the SdH oscillations can be described by the Lifshitz–Kosevich (LK) formula^38,39^:

1 $$\frac{{{\mathrm{\Delta }}\rho (T,B)}}{{\rho (B = 0)}} \propto f_m\sqrt B \frac{{2\pi ^2k_BT/\hbar \omega _c}}{{{\mathrm{sinh}}\left[ {2\pi ^2k_BT/\hbar \omega _c} \right]}}{\mathrm{exp}}\left( { - 2\pi ^2k_BT_{\mathrm{D}}/\hbar \omega _c} \right)\\ {\mathrm{cos}}\left[ {2\pi \left( {\frac{F}{B} + \gamma - \delta } \right)} \right]$$

Here, $f_m = \left| {\frac{{\partial ^2A_F}}{{\partial k_H^2}}} \right|_m^{ - 1/2}$ is an extremal curvature factor that determines the strength of the angle-dependent MR about the direction of magnetic field, *k*_*H*_ is the momentum along the magnetic field direction, *k*_*B*_ is the Boltzmann constant, *ω*_*c*_ = *eB*/m^*^ is the cyclotron frequency with m^*^the effective cyclotron mass at the Fermi energy, and *T*_D_ is the Dingle temperature. We fitted the data by the thermal damping term in the LK formula, i.e., $\frac{{2\pi ^2k_BTm^ \ast /\hbar eB}}{{{\mathrm{sinh}}\left( {2\pi ^2k_BTm^ \ast /\hbar eB} \right)}}$, with 1/*B* being the average inverse field, as shown in the inset of Fig. 2e. The determined effective masses of the two bands are $m_\alpha ^ \ast \approx 0.44 \pm 0.005\,m_0$ and $m_\beta ^ \ast \approx 0.31 \pm 0.015\,m_0$ (*m*_0_ is the free electron mass), respectively.

We extracted the oscillation components of the α and β bands as shown in the inset of Fig. 2d based on the LK formula and plotted the Landau index *n* as function of 1/*B* in Fig. 2f. For a system of *ρ*_*xx*_ ≫ *ρ*_*xy*_ as trigonal layered PtBi_2_, in presence of multibands or large MR, the maxima of resistivity should be defined as integer generally^40^. According to the phase factor of the LK formula, i.e., ${\mathrm{cos}}\left[ {2\pi \left( {\frac{F}{B} + \gamma - \delta } \right)} \right]$, where *γ* is the Onsager phase factor that is related to the Berry phase through *γ* = 1/2 – *ϕ*_*B*_/2*π*. In a conventional electron system, the Berry phase *ϕ*_*B*_ = 0, hence γ = 1/2. While for those massless Dirac materials with linear band dispersion, the nontrivial Berry phase is *ϕ*_*B*_ = *π*, which makes *γ* = 0^41^. *δ* is the phase shift determined by the dimensionality of the Fermi surface with a value equal to 0 in 2D system and ±1/8 in the 3D case. The best linear fitting of *n* vs. 1/*B* gives the intercept value of *n*_*α*_ = 0.23 ± 0.02 and *n*_*β*_ = −0.012 ± 0.05 for *α* and *β* band, respectively. The *n*_*β*_ locates between 0 ± 1/8 while *n*_*α*_ is larger than 1/8. The deviation of the intercept from the ideal value *n* = ±1/8 for both bands should be due to the reason that the Fermi level does not cross the degenerated points. However, we also note that the phase shift can be affected by spin splitting and *g*-factor, the topological nature of these two bands can not be identified just by magnetotransport measurements at present.

**Fig. 3:**
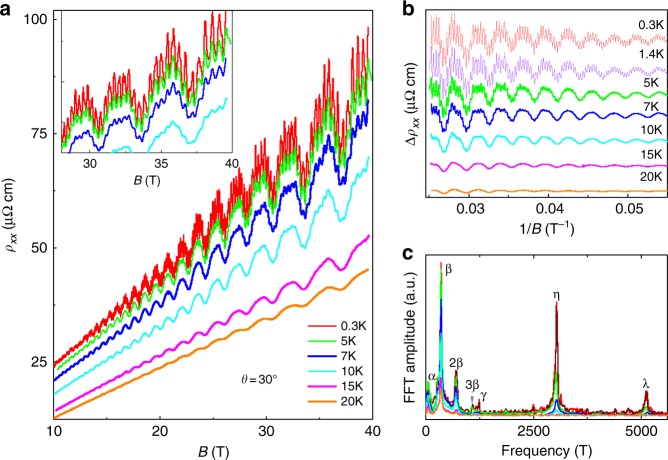
SdH oscillations in magnetic fields up to 40 T. **a** Magnetic field dependence of resistivity at different temperatures when the field is applied along *θ* = 30°. **b** SdH oscillatory components as a function of 1/*B* after subtracting a smooth background. **c** Corresponding FFT spectra of oscillations

To verify such an unsaturated MR being validated at even higher fields, we performed MR measurements on another sample S2 using a hybrid magnet with magnetic fields up to 40 T at *θ* ~ 30°, as shown in Fig. 3a. The SdH oscillations superimposed on the MR curves show more hyperfine structures than sample S1. After subtracting a three-order polynomial background, the relative oscillatory component Δ*ρ*_*xx*_ versus 1/*B* is displayed in Fig. 3(b). The FFT spectra of the oscillations as shown in Fig. 3(c) reveal five principal frequencies: *F*_*α*_ = 40 T and *F*_*β*_ = 350 T with their harmonic frequencies *F*_*2β*_ = 700 T and *F*_*3β*_ = 1050 T, *F*_*γ*_ = 1240 T, *F*_*η*_ = 3030 T, and *F*_*λ*_ = 5110 T. The lower frequencies *F*_*α*_ = 40 T and *F*_*β*_ = 350 T are the same as those of sample S1 measured with the PPMS. We determined the values of *A*_*F*_, the effective masses, and Fermi energy for the *α*, *β*, *γ*, *η*, and *λ* bands by fitting the amplitude of the SdH oscillations with the LK formula; the results are summarized in Supplementary Table 2. We have mapped the topology of the Fermi surface for the bands *α*, *β*, and *γ* through the data obtained from angle-dependent MR using a dc-resistive magnet up to 32 T on sample S1. The results suggest that the three bands show an anisotropic 3D Fermi surface, as shown in Supplementary Figs. 5 and 6.

### Magnetization oscillations

**Fig. 4:**
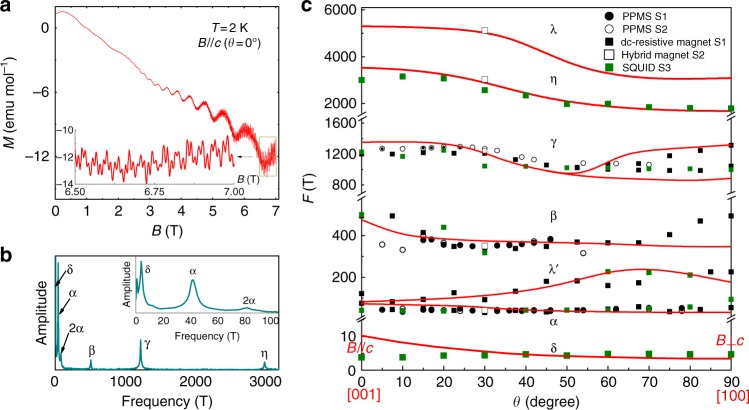
Magnetization oscillations and angle-dependent oscillation frequencies. **a** Magnetization curve measured at *T* = 2 K on sample S3 with magnetic field applied along the *c*-axis. **b** FFT spectra of dHvA oscillations. Inset: enlargement of FFT spectra. **c** Angle dependences of the frequencies extracted from the SdH and dHvA oscillations of different samples are labeled with different symbols. Theoretical calculated results are plotted with solid red lines

Figure 4a shows the magnetization versus magnetic field of sample S3 measured at *T* = 2 K by a superconducting quantum interference device (SQUID) magnetometer with a magnetic field applied along the *c*-axis. Significant dHvA oscillations are observed and can be recognized from a field as low as 1 T. Figure 4b shows the FFT spectra of the dHvA oscillations. It can be seen that, in addition to the frequencies *F*_*α*_ = 41 T, *F*_*2α*_ = 82 T, *F*_*β*_ = 510 T, *F*_*γ*_ = 1220 T, and *F*_*η*_ = 3000 T being those obtained from the SdH oscillations, an additional extreme low frequency, *F*_*δ*_ = 3.8 T, is also found. (Here, the value of band *β* is larger than that obtained from the SdH oscillations, because the field is applied along the *c*-axis, while the SdH oscillations were obtained with a field applied at *θ* = 30°.) From the temperature-dependent dHvA oscillations, the effective mass of this *δ* band was extracted to be 0.21 *m*_0_ (see Supplementary Fig. 8). Following the customary practice of defining the LL index, the *n*-versus-1/*B* curve shows a linear dependence and gives an intercept *n*_*δ*_ ~−0.48 ± 0.05. The physical parameters for the *δ* band are also included in Supplementary Table 2. Clearly, six main pockets in layered PtBi_2_ were recognized and three of them show light effective masses. In Fig. 4c, we summarize all the angle dependencies of the frequencies extracted from the SdH and dHvA oscillations of different samples. These frequencies are consistent between different samples and measurement techniques.

### Fermi surface topology

**Fig. 5:**
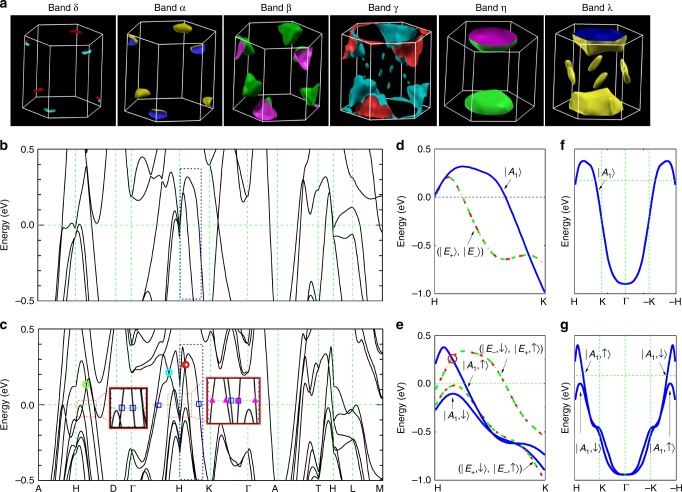
3D Fermi surface pockets and band structures. **a** Fermi surface pockets from ab initio calculations. Band structure along the direction of high-symmetry lines in the BZ (**b**) without and (**c**) spin–orbit coupling. Red circle along the H–K line labels the TP. The light blue circle along the H–Г line and the green circle along the H–D line labels the doubly degenerate points. Inset: Enlargement of the red rectangular boxes. The pink solid triangles (▲) and blue open squares (□) indicate the high-symmetry lines penetrating through the *α* and *β* pockets, respectively. **d** and **e** are magnified views of the blue-dashed rectangles in **b** and **c**, respectively. Bands are indicated by the relevant irreducible representations. **f** Spinless band along the H–K–Г–(−K)–(−H) line. **g** |A_1_〉 state split into two different substates by taking into account the spin degree of freedom due to the breaking of spatial inversion symmetry

To fully understand the transport features ascribed to unique electronic structures near the cross-points, including TPs in layered PtBi_2_, we now analyze the calculated results in detail. Figure 5a shows six bands from the ab initio calculations. It is noted that both *γ* and *λ* have several satellite pockets. The satellite pockets from the *γ* band are too tiny to be experimentally identified, while the satellite pockets from the λ band, i.e., λ′ pockets, have been identified by the transport data, as shown in the Supplementary Information. Meanwhile, the calculation result shows that the *γ* pocket has a “dumbbell” shape (see Supplementary Fig. 6). Such anisotropy can be confirmed by the angle-dependent oscillation frequency shown in Fig. 4c, in which there are two branches of the *γ* pocket. The theoretically calculated frequencies are plotted for comparison with the full angular dependence of experimental frequencies in Fig. 4c. Consistency can be observed between the two results.

### Band structures

Figure 5b, c shows the energy bands along high-symmetry lines in the BZ without and with spin–orbit coupling, respectively. The *α* and *β* pockets can be explicitly identified through the pink solid triangles (▲) and blue open squares (□), as shown in Fig. 5c. Both *α* and *β* pockets are hole type and they are linked by the TPs. The linear dispersion of the αpocket [near the red circles in Fig. 5c, e] is relevant to the TP along the H–K line, while the linear dispersion of the *β* pocket [near the light blue and green circles in Fig. 5c] is relevant to the doubly degenerate point in the mirror plane, such as the point along the H–Γ and H–D lines. Similar analyses can be applied to the *γ*, *η*, and *λ* pockets. It can be seen that theγ pocket is hole type, while the *η* and *λ* pockets are electron type, and all three pockets have quadratic dispersions with zero Berry phase. The tiny *δ* pocket also has a topologically trivial Berry phase, which is addressed in Supplementary Note 9.

## Discussion

Now, we further consider the formation of the TPs. Focusing on the band along the H–K line indicated by the blue-dashed rectangles in Fig. 5b, c, their magnified counterparts are shown in Fig. 5d, e, respectively. In the absence of spin–orbit coupling, three bands exist. Among them, two are doubly degenerate and labeled with a red-dashed line and a green dashed-dotted line, while one is onefold and labeled with a solid blue line in Fig. 5d. To avoid the complexities induced by considering the specific sublattice and hybridization between *s*, *p*, and *d* orbitals, here we use the irreducible representations of the *C*_*3v*_ point group to label the bands, i.e., |*E*_±_〉 and |*A*_1_〉. In the presence of spin–orbit coupling in Fig. 5e, the two degenerate |*E*_±_〉 states split into two doublets, i.e., (|*E*_+_,↑〉, |*E*_−_,↓〉) and (|*E*_+_,↓〉, |*E*_−_,↑〉). Such splitting is induced by the spin–orbit coupling. The |*A*_1_ state also splits into two states, i.e., |*A*_1_,↓〉 and |*A*_1_,↓〉 [as shown in Fig. 5g] when spin degrees of freedom are taken into account. Note that this splitting is induced by the absence of spatial inversion symmetry, and such splitting is similar to the splitting from Rashba spin–orbit coupling. Figure 5f, g clearly illustrates the splitting of the |*A*_1_〉 state induced by the absence of spatial inversion symmetry. The TP along the H–K lines is formed by the crossings between the doublets (|*E*_+_,↑〉, |*E*_−_,↓〉) and the singlet |*A*_1_,↑〉, while the TP along the (−H)–(−K) lines is formed by the crossings between the doublets (|*E*_+_,↓〉, |*E*_−_,↑〉), and singlet |*A*_1_,↓〉. The two TPs can transform into each other via the time-reversal-symmetry.

Finally, we point out the unique features of the TPs in trigonal layered PtBi_2_ in comparison with the TPs in WC-type materials. In the (001) surface BZ in Fig. 1d, we find that a pair of TPs is projected into two $\bar K_1$ and $\bar K_2$ points. The large separation of the two $\bar K_1$ and $\bar K_2$ points leads to the Fermi arcs that link the individual TPs on the surface and make them easily observable. In MoP, two TPs at the Γ-A line are projected onto the same Γ point, resulting in the disappearance of the Fermi arcs. Thus, the trigonal layered PtBi_2_ may be a candidate for the direct ARPES verification of TP fermions. Another remarkable feature is that the TPs in trigonal layered PtBi_2_ are quite close to the Fermi level, which provides possibilities to explore the properties of TP fermions.

In conclusion, we theoretically predict the existence of TP fermions in trigonal layered PtBi_2_. To verify their existence and explore their properties, we studied the quantum transport behaviors of high-quality trigonal layered PtBi_2_ single crystals systematically. When the magnetic field is tilted from the [001] to [100] direction, a giant linear magnetoresistance (1.3 × 10^5^% at 32 T and 2 K) without any sign of saturation at approximately θ ~ 30°, accompanied by SdH oscillations, is observed. Our analyses of the SdH and dHvA oscillations revealed the existence of six principal Fermi pockets with three pockets showing light effective masses. Further analyses demonstrate that all of the main experimental results can be well understood in the context of the electronic structures around the TP fermions. The consistencies between the theoretical and experimental results support the emergence of TP fermions in trigonal layered PtBi_2_.

## Methods

### Single-crystal preparation

The single crystals of PtBi_2_ were synthesized via the self-flux method. The raw materials with molar ratio Pt:Bi = 1:8 were put into an alumina crucible, and then the crucible was sealed into a quartz tube under vacuum. The tubes were heated up to 800 °C, dwelled for 24 h, and then slowly cooled to 430 °C a rate of 2 °C/h. At this temperature, the flux was separated by a centrifuge.

### Magnetotransport and magnetization measurements

The low-field MR was measured with standard four-probe using a 16 T PPMS (Quantum Design, Inc.). The magnetization measurements were carried out using a SQUID magnetometer. The MR at high magnetic fields (~32 and 40 T) were measured using standard a.c. lock-in techniques in either a He-3 cryostat on a dc-resistive magnet or a hybrid magnet at the high magnetic field laboratory (HMFL), Hefei, China.

### Band structure calculations

The density functional theory^42,43^ calculations were performed by using the Vienna ab initio simulation package^44^, with Perdew–Burke–Ernzerhof^45^ type generalized gradient approximation in the exchange-correlation functional. The spin–orbit coupling was taken into account. The Brillouin zone is sampled by 12 × 12 × 13 k-point mesh. The Fermi surface is obtained by using Wannier 90 package^46^.

### Data availability

The data that support the finding of this study are available from the corresponding authors upon reasonable request.

## Electronic supplementary material

Supplementary Information

## References

[1] Young SM (2012). Dirac semimetal in three dimensions. Phys. Rev. Lett..

[2] Wang Z (2012). Dirac semimetal and topological phase transitions in A_3_Bi (A = Na, K, Rb). Phys. Rev. B.

[3] Liu ZK (2014). Discovery of a three-dimensional topological Dirac semimetal, Na_3_Bi. Science.

[4] Xu SY (2015). Observation of Fermi arc surface states in a topological metal. Science.

[5] Wan XG (2011). Topological semimetal and Fermi-arc surface states in the electronic structure of pyrochlore iridates. Phys. Rev. B.

[6] Burkov AA (2011). Topological nodal semimetals. Phys. Rev. B.

[7] Weng H (2015). Weyl semimetal phase in noncentrosymmetric transition-metal monophosphides. Phys. Rev. X.

[8] Huang SM (2015). A Weyl fermion semimetal with surface Fermi arcs in the transition metal monopnictide TaAs class. Nat. Commun..

[9] Xu SY (2015). Discovery of a Weyl fermion semimetal and topological Fermi arcs. Science.

[10] Soluyanov AA (2015). Type-II Weyl semimetals. Nature.

[11] Liu ZK (2014). A stable three-dimensional topological Dirac semimetal Cd_3_As_2_. Nat. Mater..

[12] Borisenko S (2014). Experimental realization of a three-dimensional Dirac semimetal. Phys. Rev. Lett..

[13] Madhab N (2014). Observation of a three-dimensional topological Dirac semimetal phase in high mobility Cd_3_As_2_. Nat. Commun..

[14] Lu L (2015). Experimental observation of Weyl points. Science.

[15] Lv BQ (2015). Observation of Weyl nodes in TaAs. Nat. Phys..

[16] Gábor. BH (2012). Time-reversal invariant realization of the Weyl semimetal phase. Phys. Rev. B.

[17] Burkov AA (2011). Weyl semimetal in a topological insulator multilayer. Phys. Rev. Lett..

[18] Benjamin JW (2016). Double Dirac semimetals in three dimensions. Phys. Rev. Lett..

[19] Barry B (2016). Beyond Dirac and Weyl fermions: unconventional quasiparticles in conventional crystals. Science.

[20] Weng H (2016). Coexistence of Weyl fermion and massless triply degenerate nodal points. Phys. Rev. B.

[21] Weng H (2016). Topological semimetals with triply degenerate nodal points in *θ*-phase tantalum nitride. Phys. Rev. B.

[22] Zhu Z (2016). Triple point topological metals. Phys. Rev. X.

[23] Chang G (2017). Nexus fermions in topological symmorphic crystalline metals. Sci. Rep..

[24] Lv BQ (2016). Observation of three-component fermions in the topological semimetal molybdenum phosphide. Nature.

[25] Ma JZ (2018). Three-component fermions with surface Fermi arcs in tungsten–carbide. Nat. Phys..

[26] Shekhar, C. et al. Extremely high conductivity observed in the unconventional triple point fermion materials MoP. Preprint at https://www.arxiv.org/abs/1703.03736 (2017).

[27] He JB (2017). Magnetotransport properties of triply degenerate node topological semimetal tungsten–carbide. Phys. Rev. B.

[28] Zhu, W. L. et al. Magnetotransport properties of the new-type topological semimetal ZrTe. Preprint at https://www.arxiv.org/abs/1707.00942 (2017).

[29] Martin K (2014). Bi_2_Pt (hP9) by Low-temperature reduction of Bi_13_Pt_3_I_7_: reinvestigation of the crystal structure and chemical bonding analysis. Z. Anorg. Allg. Chem..

[30] Xu CQ (2016). Synthesis, physical properties, and band structure of the layered bismuthide PtBi_2_. Phys. Rev. B.

[31] Yang XJ (2016). Giant linear magnetoresistance in nonmagnetic PtBi_2_. Appl. Phys. Lett..

[32] Du X (2005). Metal-insulator-like behavior in semimetallic bismuth and graphite. Phys. Rev. Lett..

[33] Mazhar NA (2014). Large, nonsaturating magnetoresistance in WTe_2_. Nature.

[34] Chandra S (2015). Extremely large magnetoresistance and ultrahigh mobility in the topological Weyl semimetal candidate NbP. Nat. Phys..

[35] Tafti FF (2016). Resistivity plateau and extreme magnetoresistance in LaSb. Nat. Phys..

[36] Xiong J (2015). Evidence for the chiral anomaly in the Dirac semimetal Na_3_Bi. Science.

[37] He LP (2014). Quantum transport evidence for the three-dimensional Dirac semimetal phase in Cd_3_As_2_. Phys. Rev. Lett..

[38] Murakawa H (2013). Detection of Berry’s phase in a bulk Rashba semiconductor. Science.

[39] Lifshits IM, Kosevich AM (1956). On the theory of magnetic susceptibility in metals at low temperatures. Sov. Phys. JETP.

[40] Ando Y (2013). Topological insulator materials. J. Phys. Soc. Jpn..

[41] Xiao D, Chang MC, Niu Q (2010). Berry phase effects on electronic properties. Rev. Mod. Phys..

[42] Hohenberg P (1964). Inhomogeneous electron gas. Phys. Rev..

[43] Kohn W (1965). Self-consistent equations including exchange and correlation effects. Phys. Rev..

[44] Kresse G (1996). Efficient iterative schemes for ab initio total-energy calculations using a plane-wave basis set. Phys. Rev. B.

[45] John PP (1996). Generalized gradient approximation made simple. Phys. Rev. Lett..

[46] Arash AM (2014). An updated version of Wannier 90: a tool for obtaining maximally localised Wannier functions. Comput. Phys. Commun..

