# Supplementary material for: A possible candidate for triply degenerate point fermions in trigonal layered PtBi2
Source: Nat Commun. 2018 Aug 14;9:3249. doi: 10.1038/s41467-018-05730-3 (PMC6092399; doi:10.1038/s41467-018-05730-3)
Supplement: Supplementary file 1 — Supplementary Information [file 41467_2018_5730_MOESM1_ESM.pdf]

Supplementary Information

A possible candidate for triply degenerate point fermions in trigonal layered $PtBi_2$

Gao et al.

Supplementary Note 1. Crystal structure of trigonal layered $PtBi_2$.

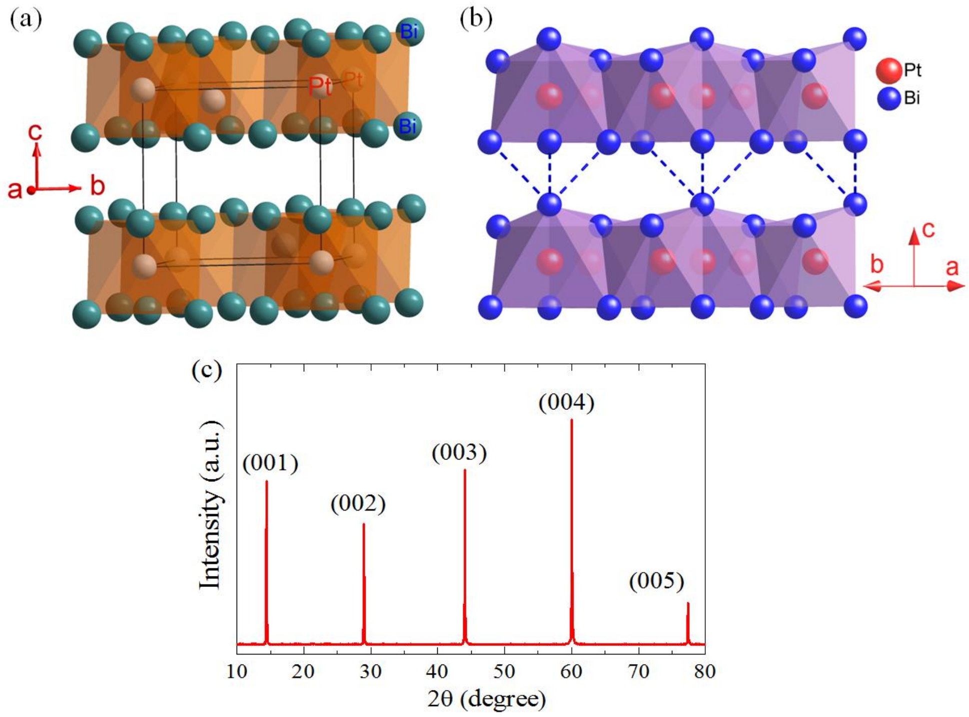

Supplementary Figure 1 | Crystal structure of trigonal layered $PtBi_2$. (a) The crystal structure of layered $PtBi_2$ without lattice distortion (space group $P\bar{3}$). (b) The crystal structure of layered $PtBi_2$ with lattice distortion (space group P31m). (c) X-ray diffraction pattern of a single crystal on the (00L) plane.

Supplementary Figure 1(a) shows the crystal structure of layered $PtBi_2$ without lattice distortion (space group $P\bar{3}$). The Pt layers are separated by two neighboring Bi layers, where both are sandwiched between Pt atoms. All these layers are situated in the (*a*,*b*) plane and van der Waals stacked along the crystallographic *c*-axis of the trigonal unit cell, forming a layered crystal structure. Supplementary Figure 1(b) shows the crystal structure of layered $PtBi_2$ with lattice distortion(space group *P*31*m*). Both top and bottom Bi layers shown in the figure are distorted.

Supplementary Figure 1(c) shows the x-ray diffraction (XRD) spectra of a typical single crystal. Sharp (00L) reflections indicate high crystallinity of these crystals, which also suggests that the exposed surface of $PtBi_2$ crystal belongs to (*a*,*b*) plane. Note that the distortion of Bi layer is quite small. The XRD cannot identify such tiny distortion.

We have performed the *ab initio* calculations with the Crystal Structure Analysis by Particle Swarm Optimization (CALYPSO) algorithm to test the various low-energy structures of $PtBi_2$ compounds. The first ten low-energy structures are listed in Table I, from which we can find that the structure with space group *P*31*m* (157)

is the lowest-energy state. The calculation result is consistent with the experimental reports in Ref.[1].

Supplementary Table 1. The first ten low-energy structures obtained from *ab initio* calculations with Crystal Structure Analysis by Particle Swarm Optimization (CALYPSO) algorithm

No.	Enthalpy	Space group
1	-4.82813	P31m(157)
2	-4.81830	R-3m(166)
3	-4.81688	P-1(2)
4	-4.80955	P-1(2)
5	-4.80829	P-1(2)
6	-4.80762	P-3m1(164)
7	-4.80255	C2/m(12)
8	-4.80001	Cm(8)
9	-4.79798	P3_221(154)
10	-4.77954	P-1(2)

Supplementary Note 2.Anisotropic magnetoresistance (AMR) in layered $PtBi_2$.

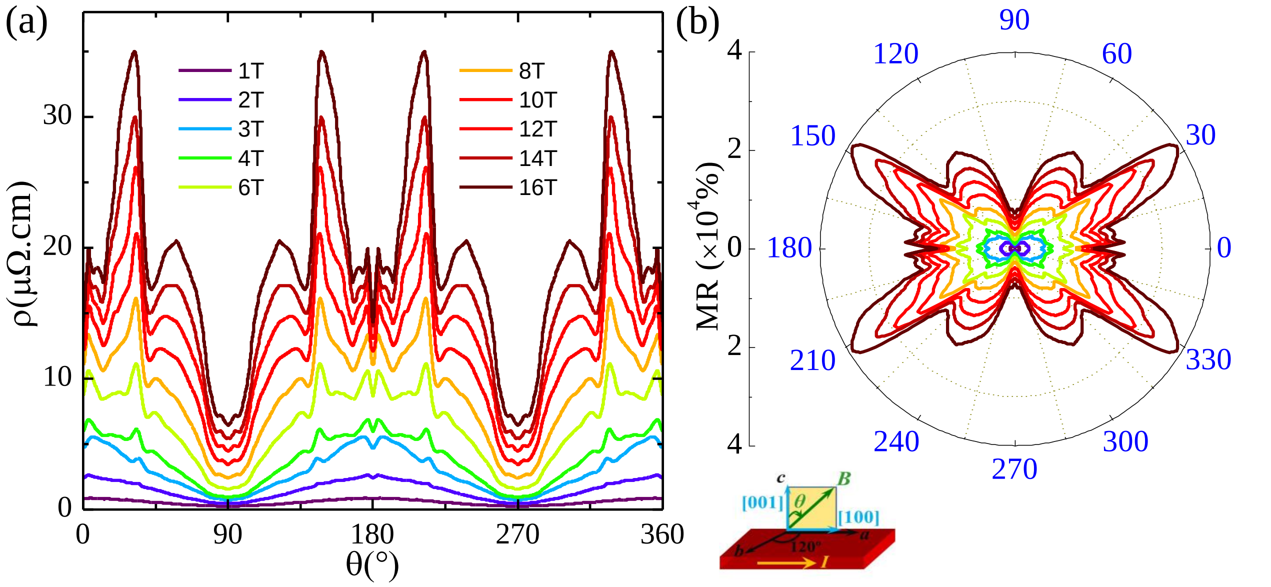

Supplementary Figure 2 | Anisotropic magnetoresistance (AMR) in layered $PtBi_2$. (a) Resistivity of $PtBi_2$ as a function of θ under different magnetic fields. **(b)** The polar diagram of AMR under different magnetic fields.

Supplementary Figure 2(a) shows the angle-dependent resistivity of sample S1 measured at 2 K with different magnetic fields tilted within (a,c) plane. Figure S2(b) shows the polar plot of anisotropic magnetoresistance (AMR). It is clearly that the MR reaches the maximum with θ=30°, 150°, 210° and 330°. When the magnetic field is below ~ 1 T, the AMR curve performs a two-fold symmetry. With increasing magnetic field strength to above 2 T, the curve begins to split into more peaks, and exhibits a "butterfly" shape with texturing of complicated symmetries. In recent studies on Dirac semimetal LaBi [2] and nodal-line semimetal ZrSiS [3] and ZrSiSe [4], four-leaf-like MR behavior has been observed, but the butterfly pattern with much higher order texturing has never been observed before in other topological semimetals and might be associated with the complicated multiple band structures in layered $PtBi_2$.

Supplementary Note 3. Phase factors of α and β bands.

To detect the band topology, actually, we can also verify the phase factor through the direct fit of the oscillation pattern to the Lifshitz-Kosevich (LK) formula.

$$\frac{\Delta\rho}{\rho(B=0)} \propto f_m\sqrt{B}\frac{2\pi^2 k_B T m^*/\hbar eB}{\sinh(2\pi^2 k_B T m^*/\hbar eB)} e^{-2\pi^2 k_B T_D m^*/\hbar eB} \cos\left[2\pi\left(\frac{F}{B}+\gamma-\delta\right)\right] , \quad (1)$$

Here, $f_m = \left|\frac{\partial^2 A_F}{\partial k_H^2}\right|_m^{-1/2}$ is the extremal curvature factor, which determines the strength of the angular-dependent MR about the direction of magnetic field. k_H is the momentum along the magnetic field direction. k_B is the Boltzmann constant, $\hbar$ is the Planck's constant, m^* is the effective cyclotron mass at the Fermi energy and T_D is the Dingle temperature, γ is the Onsager phase factor that is related to the Berry phase, δ is the phase shift determined by the dimensionality of the Fermi surface with a value equal to 0 in 2D system and ±1/8 in the 3D case. The fitting was performed using the multiband LK formula for which the multiple frequency oscillations are treated as linear superposition of several single-frequency oscillations. With the effective masses, frequencies, Dingle temperature and the phase factor (intercepts $n_\alpha = 0.23$ and $n_\beta = 0.02$) as the fixed parameters, we expect to duplicate the oscillation patterns through adjusting the proportionality coefficient of two frequencies, as shown in Supplementary Fig. 3, the multiband LK model reproduces the oscillation patterns very well.

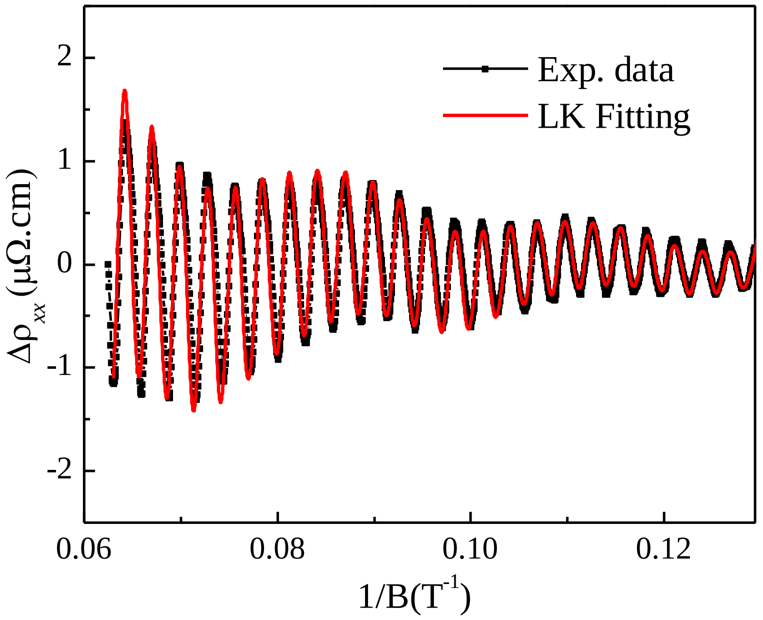

Supplementary Figure 3 | The multiband Lifshitz-Kosevich (LK) fit (red line) of the SdH oscillation pattern (black points) when the field is applied along θ = 30° at T=2K.

Supplementary Note 4. Physical parameters for the six principle pockets extracted from the SdH and dHvA oscillations.

The FFT spectra of sample S2 (Fig.3(c) in the main text) were obtained from the magnetic field range of 10 ~ 40 T, then we extracted the FFT amplitude of different temperatures and fitted the data using the thermal damping term in the LK formula, *i.e.*, $\frac{2\pi^2 k_B T m^*/\hbar eB}{sinh\,(2\pi^2 k_B T m^*/\hbar eB)}$ with $1/B$ being the average inverse field. The determined effective masses of the five bands are $m_\alpha^* \approx 0.30 \pm 0.01\, m_0$, $m_\beta^* \approx 0.34 \pm 0.01\, m_0$, $m_\gamma^* \approx 0.82 \pm 0.02\, m_0$, $m_\eta^* \approx 0.96 \pm 0.01\, m_0$, $m_\lambda^* \approx 0.99 \pm 0.03\, m_0$ (m_0 is the free electron mass), respectively. We note that here the magnetic field range 10 ~ 40 T is larger than that of sample S1 (8-16T). The higher field range of S2 might leads to an underestimation of the effective mass for different bands.

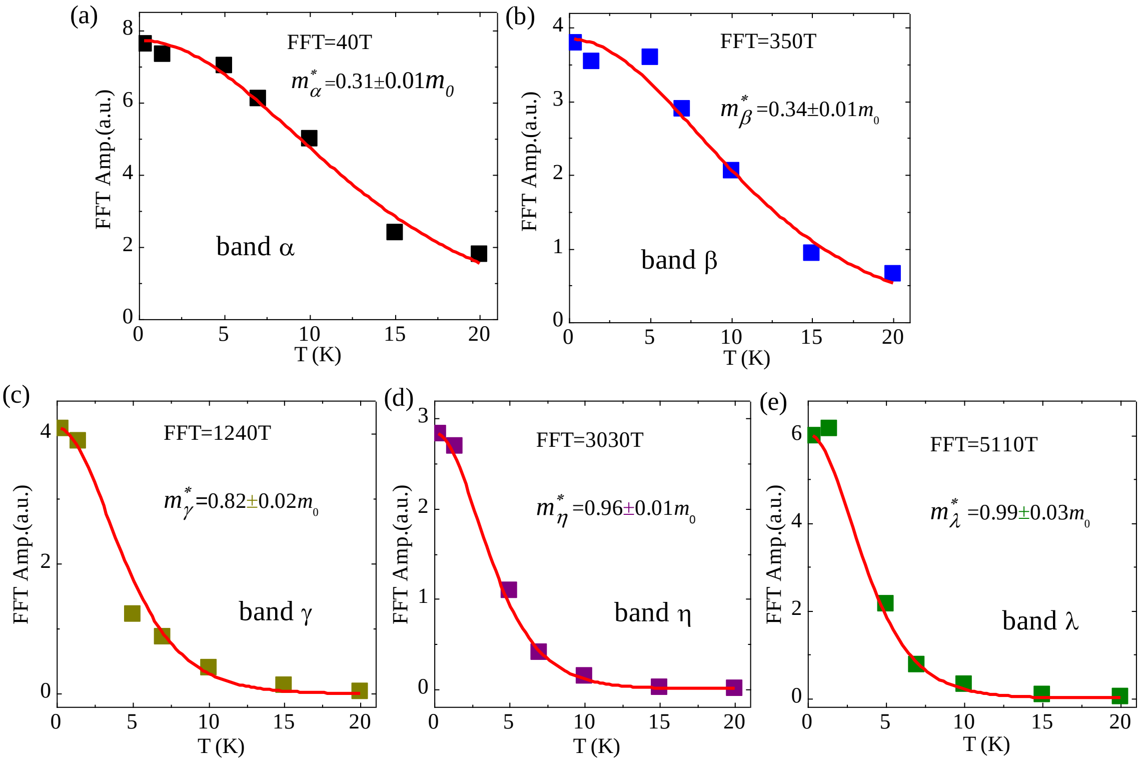

Supplementary Figure 4 | Effective masses of different pockets extracted from the data of sample S2 in magnetic fields up to 40 T. The solid red curves are the fits to the LK formula.

Supplementary Table 2. Physical parameters for the six Fermi pockets.

	θ=0°(dHvA)	θ=30 ±5°(SdH)						
Pockets	δ (S3)	α (S1)	β(S1)	α(S2)	β(S2)	γ(S2)	η(S2)	λ(S2)
F(T)	3.8	40	350	40	350	1240	3030	5110
	(10)	(45)	(375)			(1215)	(2960)	(5060)
S_F(Å^{-2})	0.0003(6)	0.003(8)	0.033(1)	0.003(8)	0.033(1)	0.117(7)	0.287(8)	0.485(4)
k_F(Å^{-1})	0.010(7)	0.034(7)	0.102(7)	0.034(7)	0.102(7)	0.193(6)	0.302(3)	0.393(1)
m^*(m_0)	0.21(4)	0.44(1)	0.31(1)	0.30(1)	0.34(1)	0.82(2)	0.96(1)	0.99(3)
v_F(10^5m/s)	0.59(4)	0.913(5)	3.836(5)	1.33(5)	3.499(2)	2.727(5)	3.646(7)	4.584(4)
E_F(meV)	4.16(4)	20.85(6)	259.40(2)	30.40(4)	236.73(8)	347.74(2)	726.74(0)	1186.77(9)

Supplementary Table 2 shows the physical parameters of the six pockets through fitting the SdH and dHvA oscillations of three samples（S1,S2 and S3）. Here the dHvA oscillations of sample S3 were obtained with the field applied along c-axis, while the SdH oscillations of sample S1 and S2 were obtained with a field applied around θ =30°. The second row of F(T) is the calculated result for each band.

Supplementary Note 5. Angle-dependent MR of sample S1 with magnetic field up to 32T

We performed the transport measurement on sample S1 in a dc-resistive magnet with magnetic fields up to 32T. As shown in Supplementary Fig. 5(a), the MR shows the sample angle dependence, which is similar with those obtained from PPMS. For example, the MR presents the largest unsaturated MR (1.3×10^5% at 32T and 2K) at θ=30°. The amplitude of the SdH oscillations is also the largest around θ=30°. The FFT spectra with θ=30° was shown in Supplementary Fig. 5(b). Compared with the result measured with PPMS, an additional frequency F_γ = 1210 T is found.

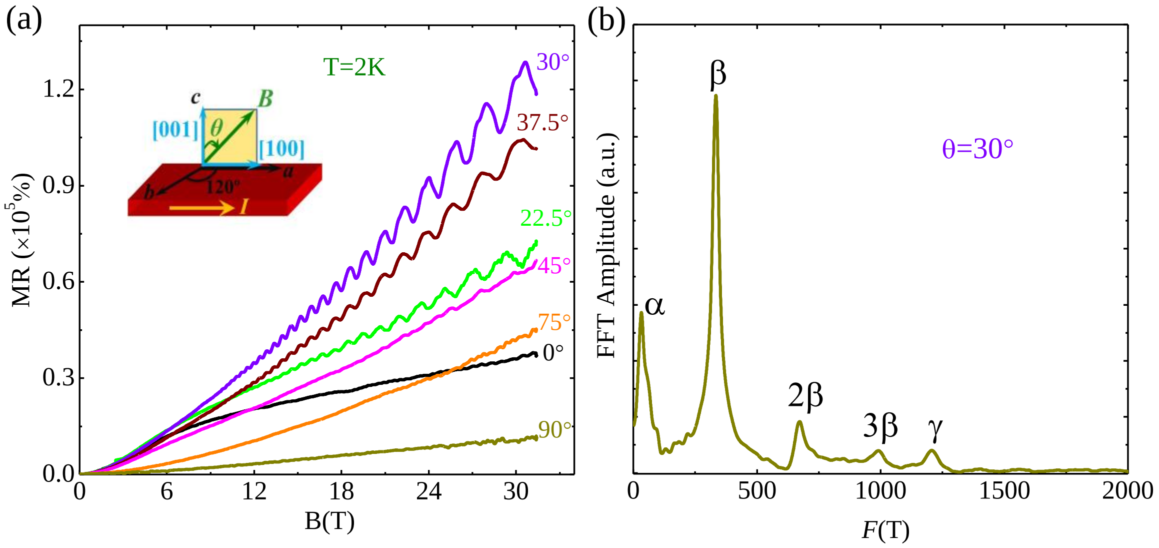

Supplementary Figure 5 | MR versus *B* of sample S1 in the field range of 32 T. (a) MR with different magnetic field orientations at T=2 K. (b) The corresponding FFT spectra of the SdH oscillation at θ=30°.

Supplementary Note 6. Topology of Fermi surfaces in $PtBi_2$.

After subtracting three-order polynomial background of the MR curves in Supplementary Fig.5(a)，the oscillatory component as a function of 1/*B* were shown in Supplementary Figure 6 (a). Besides the α , β and γ pockets identified with PPMS measurement on S1, a new pocket with the frequency near α pocket, labeled λ’, can be recognized, as shown in Supplementary Fig. 6(b). Additionally, it can be noted that the angular dependence of the oscillation frequency for γ pocket has two branches. Such a frequency splitting should be related to the “dumbbell” shape of this pocket, as shown in Supplementary Fig. 6 (c). All the angular dependence of frequencies were summarized in the Fig.4(c) in the main text and were consistent with theoretical calculated results.

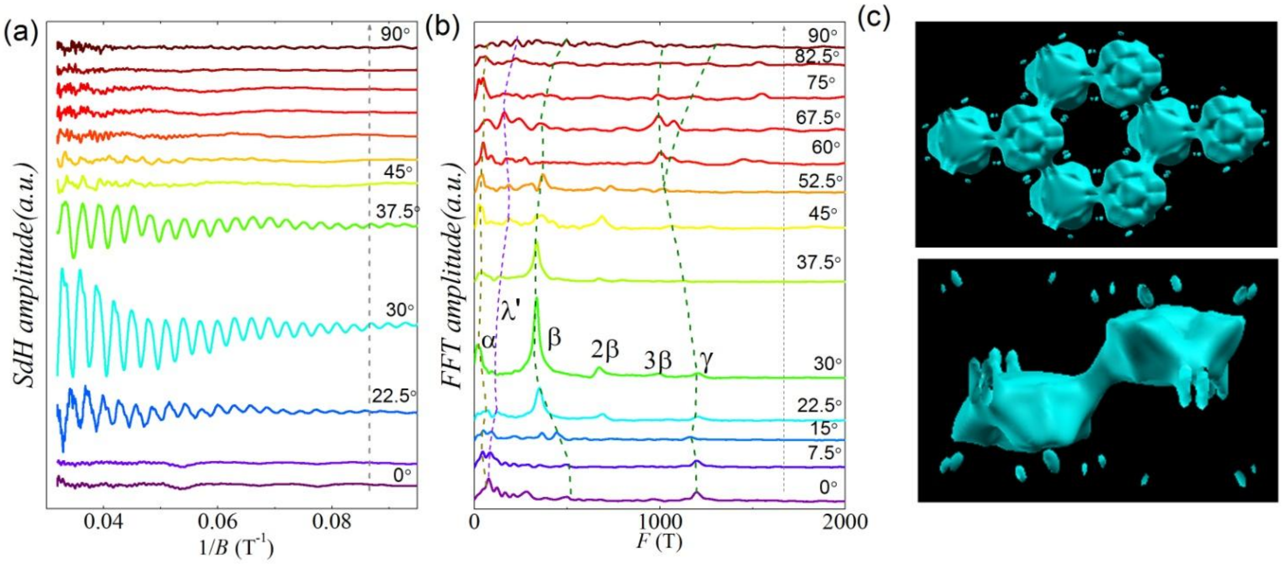

Supplementary Figure 6 | Fermi surface topology of $PtBi_2$ probed by angle-dependent MR measurements. (a) The SdH oscillations as a function of 1/*B* at different angles from 0° to 90°. **(b)** The FFT spectra obtained from the SdH oscillations at different angles. **(c)** The Fermi surface of band γ. Top and bottom patterns represent top and side view, respectively.

Supplementary Note 7. The Hall resistivity ρ_{xy} of $PtBi_2$

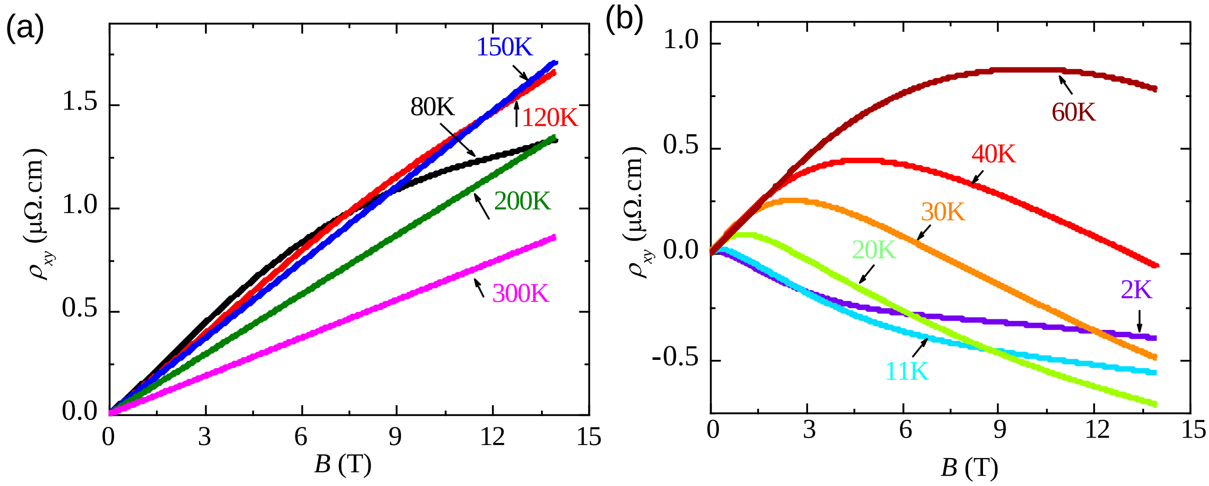

Supplementary Figure 7| The Hall resistivity ρ_{xy} obtained at several temperatures for sample S1. (a) Hall resistivity above 80 K. (b) Hall resistivity in the temperature range of 2-60K.

Supplementary Figure 7 presents the field dependence of Hall resistivity ρ_{xy} at several temperatures for sample S1. At high temperatures, ρ_{xy} exhibits linear field dependence with positive slope, suggesting the hole carriers are dominant. As temperature decreases below 80 K, ρ_{xy} keeps positive slope at low fields but changes to negative at higher fields, implying the coexistence of two types of carriers. At low temperatures below 20 K, the

Hall data suggests that more than two bands contribute to the transport properties. These results further confirm that $PtBi_2$ is a complicated multiband system, which is consistent with the theoretical calculations of layered $PtBi_2$.

Supplementary Note 8. The dHvA oscillations at different temperatures

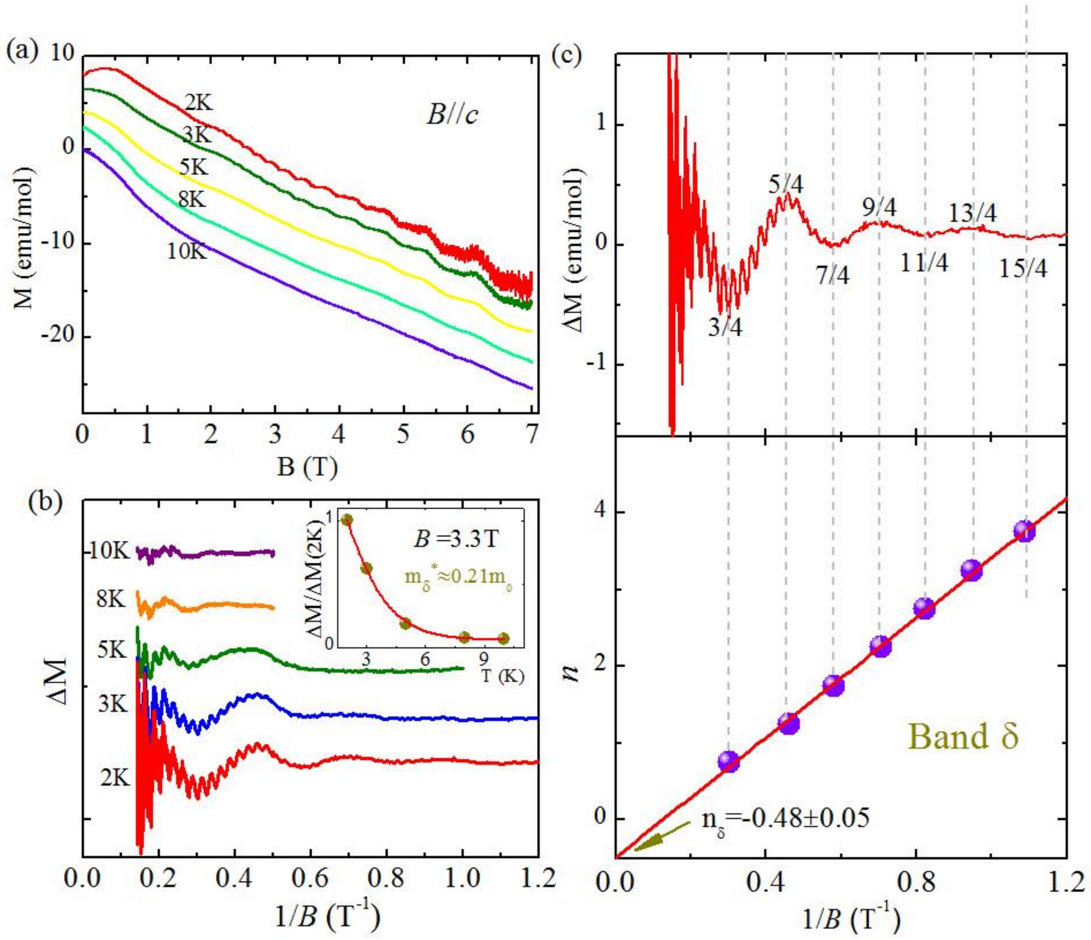

Supplementary Figure S8| Magnetization oscillations in magnetic field up to 7T. (a) Magnetization curves measured at different temperatures on sample S3 with magnetic field applied along *c*-axis. (b) The dHvA oscillations versus 1/B at different temperatures after subtracting the background. Inset: The temperature dependence of the FFT amplitude. The solid curve is the fit to the LK formula. (c) The Landau level indices *n* of δ-band extracted from the dHvA oscillations are plotted as a function of 1/*B*.

Supplementary Figure 8(a) shows dHvA oscillations at different temperatures with magnetic field applied parallel to the c-axis (θ =0°). After subtracting a three-order polynomial background, the relative oscillatory component ΔM versus $1/B$ was displayed in Supplementary Fig. 8(b). The effective mass of δ-band was extracted to be 0.21 m_0, as shown in the inset of Supplementary Fig. 8(b). Following the customary practice of defining the LL index, a minimum in conductivity $\Delta\sigma_{xx} \sim 1/\Delta\rho_{xx}$ is associated with the integer LL indices n. According to the theoretical relation, the oscillatory magnetic susceptibility $d(\Delta M)/dB \sim \Delta\sigma_{xx}$, and the minima of

ΔM are shifted by $\pi/2$ in comparison to the minima of magnetic susceptibility and conductivity, thus the minima of ΔM should be assigned to n-1/4 [5]. As illustrated in Supplementary Fig. 8(c), the index number n versus $1/B$ curve shows a linear dependence and gives an intercept n_δ=-0.48±0.05, which is close to -1/2.

Supplementary Note 9. The angular dependence of the dHvA oscillations measured at 2K.

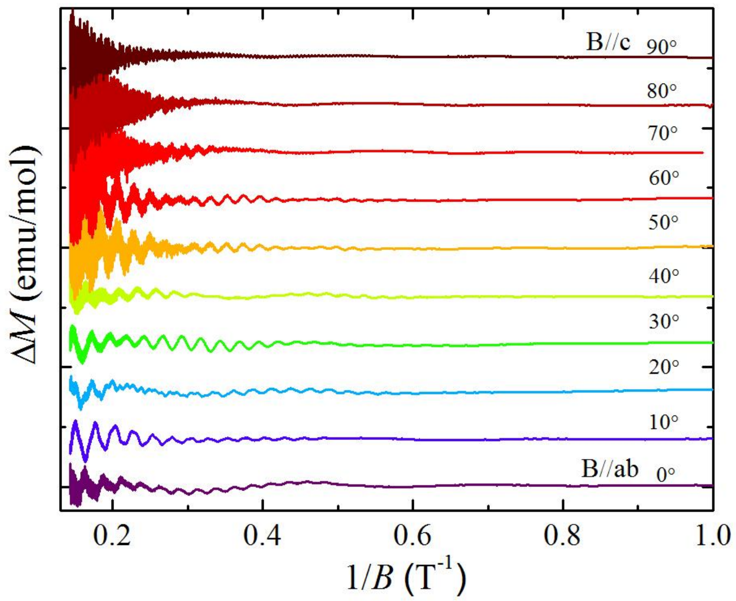

Supplementary Figure 9| The dHvA oscillations at various angles plotted as a function of 1/B.

Supplementary Figure 9 shows the angular dependence of the dHvA effect when field oriented from c-axis to ab-plane. The corresponding oscillations of low frequency δ band were observed throughout the angle region, which help us to establish a 3D Fermi surface of δ pocket.

Supplementary Note 10. Band structure of δ band.

The δ pocket can be visualized by plotting the bands along some non-high-symmetry lines. The band along the (H-) - (A-) direction with k_z=0.2 |HK| is shown in Supplementary Figs. 10 (a) and (b) without and with spin-orbital coupling. The three bands can also be characterized with $|E_\pm\rangle$ and $|A_1\rangle$ without spin-orbit coupling at H- point. Upon the spin-orbital coupling, the three bands split into six bands as shown in Supplementary Fig. 10 (b). In comparison with Supplementary Fig. 10 (a), we can find that the arrangement orders of bands according to energy switch from $|A_1\rangle$, $|E_\pm\rangle$ to $|A_1,\uparrow\rangle$, $|A_1,\downarrow\rangle$, $|E_\pm,\uparrow\rangle$ and $|E_\pm,\downarrow\rangle$. It means the band inversion occurring at H- point. Furthermore, apart from H- point and along the (H-) - (A-) line, the split $|A_1\rangle$ band crosses with one split band from $|E_\pm\rangle$ to open a band gap. That is the standard process of topological phase transition. We can announce that the small gap near H-point is topological nontrivial. However, the situation is very subtle. From the calculation, the

Fermi level is very close to the gap edge but does not cross the nontrivial band gap. Therefore, the δ pocket is topologically trivial from these ideal calculated results. Actually, several perturbations can dramatically change the topological features of the pocket. For example, the unavoidable impurities can slightly move the Fermi level up or down to give the trivial or nontrivial case.

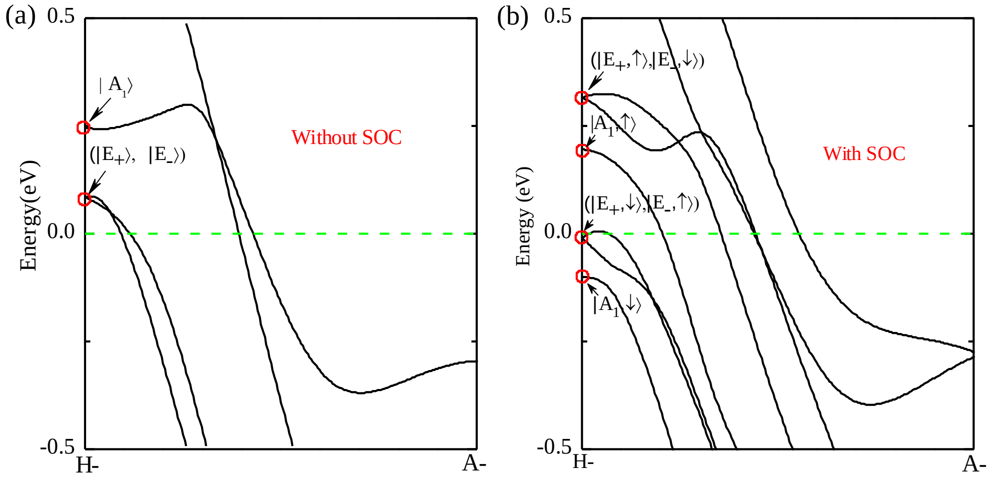

Supplementary Figure S10| The band structures along the (H-) - (A-) direction with k_z=0.2|HK| without spin-orbital coupling in (a) and with spin-orbital coupling in (b).

Supplementary Note 11. Understanding the angular dependent SdH oscillations from the anisotropy of Fermi surface.

To understand the pronounced SdH quantum oscillations around θ=30° shown in Fig. 2 (b) in the main text, we start with the LK formula in equation (1). It is noted that the curvature factor $f_m = \left|\frac{\partial^2 A_F}{\partial k_H^2}\right|_m^{-1/2}$ has close relation with the angular-dependent MR oscillation when the direction of magnetic field rotates. The angular-dependent relation of MR oscillations is dominated by the curvature factor f_m. Furthermore, the pronounced SdH quantum oscillation is mainly from a superposition of the oscillations of α and β pockets, as shown in Fig. 2 (d) and Supplementary Fig. 6 (a). Therefore, we focus on these two pockets. In Supplementary Figs. 11 (b) and (c), we plot the cross section A_F *vs* k_H. To calculate f_m, we use the quadratic polynomial to fit the $A_F(k_H)$. Such selection is due to the fact that the general energy dispersion has the quadratic about k_H i.e., E $\propto (ak_{\parallel}^2 + bk_H^2)$. The cross section $A_F(k_H) \sim \pi k_{\parallel}^2 \propto (E - bk_H^2)$. Thus, the f_m is a constant under such approximation. The calculated results are shown in Supplementary Fig. 11 (d), and several features can be found. First, the curvature factors at titled angle 30° are larger than these at titled angle 0° for both pockets. Second, the curvature factor for β pocket is larger than that for α pocket at the same titled angle. As a consequence, the oscillations from β pocket is primary while the oscillation from α pocket is secondary. The overall SdH oscillation pattern has same

angular-dependent relation as that of β pocket. All in all, the pronounced SdH quantum oscillation at some special angle can be understood with aforementioned approximation and calculation.

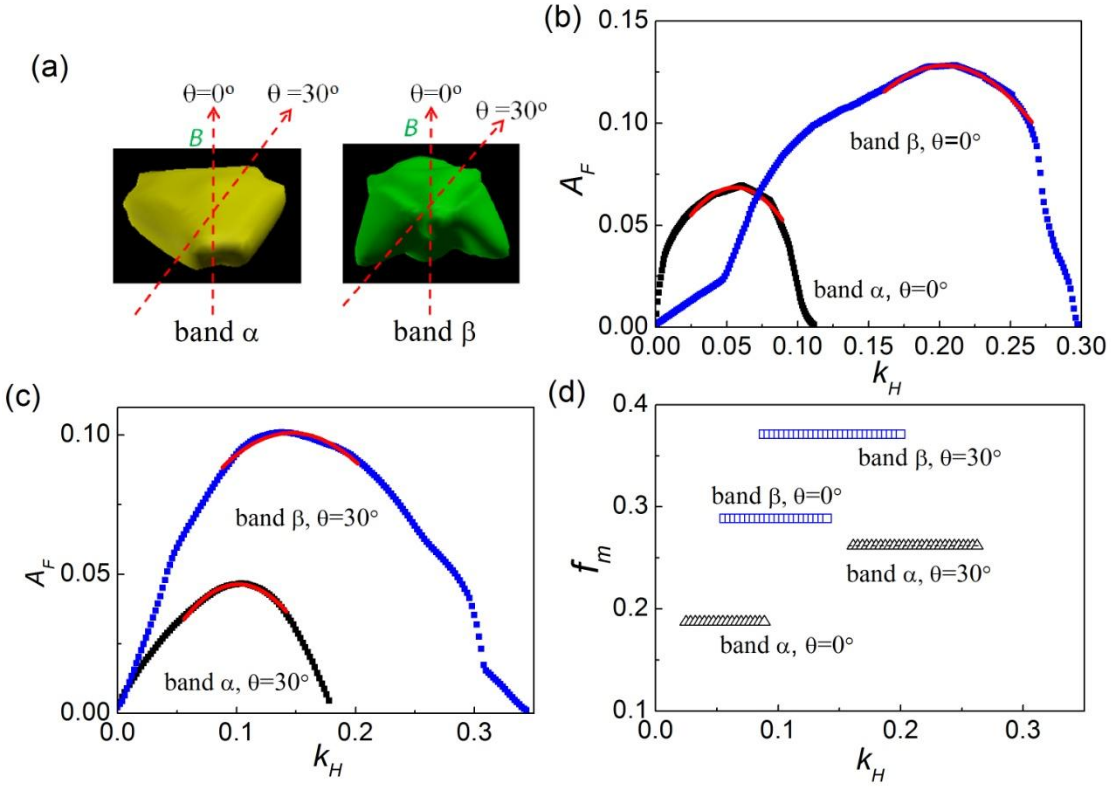

Supplementary Figure. 11| Fermi surface topology of α and β bands and curvature factor. (a) The closed Fermi surface for α and β pockets. (b) and (c) are the cross sections of α and β pockets *vs* the momentum k_H along the direction of magnetic field. Here, the titled angle is 0° in (b) and 30° in (c). The dotted and solid lines represent the calculated and fitting results, respectively. (d) The curvature factor f_m for α and β pockets with titled angles 0° and 30°.

Supplementary Note 12. The electronic structures of the non-distortion $PtBi_2$

As a reference structure of layered trigonal $PtBi_2$, the non-distortion $PtBi_2$ provides useful information to further testify the validity of such distortion from the electronic structures in comparison with the experimental observation. The crystalline and electronic structures of non-distortion $PtBi_2$ are shown in Supplementary Fig. 12. From Supplementary Figs. 12 (e)-(j), one can find that there exist six Fermi pockets. However, the pockets in Supplementary Figs. 12 (e) and (f) are degenerate, and the pockets in Supplementary Fig. 12 (i) and (j) are also degenerate. The pockets in Supplementary Figs. 12 (i) and (j) is connected by mirror transformation m_z. Thus, the transport experiment cannot distinguish the pockets in Supplementary Figs. 12 (g) and (h).. In this case, there should be three but not six pockets identified by the transport experiment if the sample has the non-distortion structure.

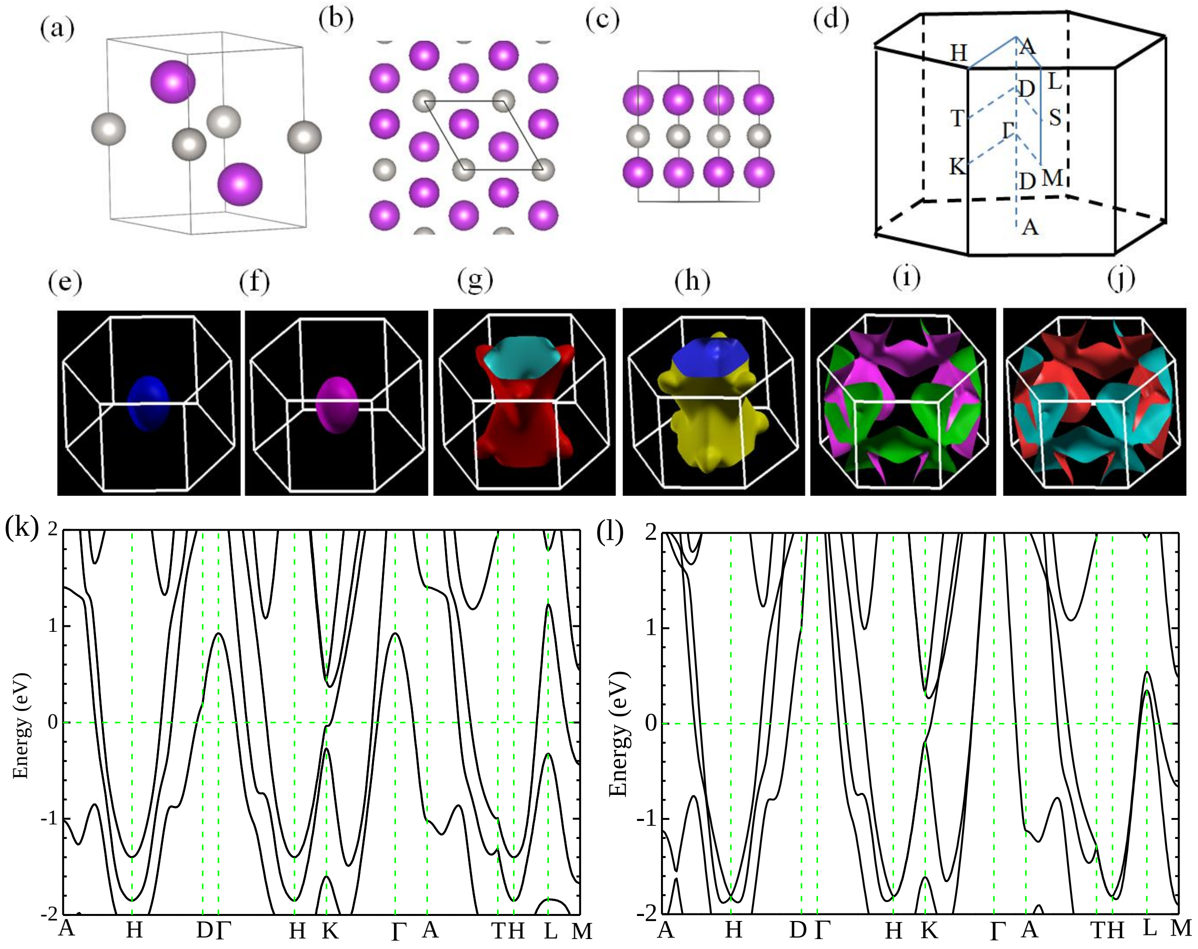

Supplementary Figure 12 | Crystal structure and calculated electron structures of non-distortion $PtBi_2$. (a) The crystalline structure of non-distortion $PtBi_2$. (b) The top view of (a). (c) the side view of (a). (d) Brillouin zone of non-distortion $PtBi_2$. (e)-(j) the six Fermi pockets. (k) and (l) the band structures of non-distortion $PtBi_2$ without and with spin orbit coupling, respectively.

Supplementary References

[1] Kaiser, M. *et al.* Bi_2Pt (hP9) by Low-temperature reduction of $Bi_{13}Pt_3I_7$: reinvestigation of the crystal structure and chemical bonding analysis. *Z. Anorg. Allg. Chem*. 640(14): 2742-2746 (2014).

[2] Nitesh, K. *et al.* Observation of pseudo-two-dimensional electron transport in the rock salt-type topological semimetal LaBi. *Phys. Rev. B* 93, 241106(R) (2016).

[3] Mazhar, N. A.*et al.* Butterfly magnetoresistance, quasi-2D Dirac Fermi surfaces, and a topological phase transition in ZrSiS. Preprint at *arXiv*: 1603.09318.

[4] Jin, H. *et al.* Evidence of topological nodal-line fermions in ZrSiSe and ZrSiTe. *Phys. Rev. Lett.* 117, 016602 (2016).

[5] Hu, J. *et al.* Nearly massless Dirac fermions and strong Zeeman splitting in the nodal-line semimetal ZrSiS probed by de Haas–van Alphen quantum oscillations. *Phys. Rev. B. 96, 045127* (2017).
